# Human-centered AI to promote youth mental health: a serendipitous natural experiment enabled by a digital health platform

**DOI:** 10.7717/peerj.20772

**Published:** 2026-02-02

**Authors:** Tarun Reddy Katapally, Nadine Elsahli, Sheriff Tolulope Ibrahim, Jasmin Bhawra

**Affiliations:** 1 DEPtH Lab, School of Health Studies, Faculty of Health Sciences, University of Western Ontario, London, Ontario, Canada; 2 Department of Epidemiology and Biostatistics, Schulich School of Medicine and Dentistry, University of Western Ontario, London, Ontario, Canada; 3 Lawson Health Research Institute, University of Western Ontario, London, Ontario, Canada; 4 CHANGE Research Lab, School of Occupational and Public Health, Ryerson Polytechnic University, Toronto, Ontario, Canada

**Keywords:** Digital citizen science, Digital transformation of health systems, Human-computer interaction, Indigenous youth health, Systems integration

## Abstract

**Background:**

Health systems are struggling to deliver timely preventive care, particularly for marginalized populations, necessitating integration across health, education, and social services. For Indigenous youth in rural communities, fragmented services, isolation, and limited culturally safe options worsen mental health disparities. Interactive technologies, particularly human-centered artificial intelligence (AI)-enabled digital health platforms grounded in human-computer interaction (HCI), can enable remote interaction with citizens and decision-makers. This study investigated a serendipitous natural experiment to assess varying levels of platform nudging on Indigenous youth compliance in a longitudinal intervention.

**Method:**

This study emerged from the final year of a 5-year initiative embedding a culturally appropriate digital health intervention into school curricula in rural Indigenous communities. While the broader aim was to assess long-term mental health outcomes, an unexpected system disruption assessment of digital nudging on compliance. The platform featured two interfaces: a citizen-facing mobile app for ecological assessments and nudges, and a scientist dashboard for monitoring engagement and triggering nudges. Youth received three nudges: (1) daily system-triggered reminders to complete assessments, (2) weekly non-personalized messages (*e.g*., land-based activity reminders), and (3) weekly personalized “Best Picture” messages showcasing youth-submitted images. The disruption created four phases: Phase 1 included all nudges; Phase 2 removed non-personalized and personalized nudges; Phase 3 reintroduced them; Phase 4 removed only personalized nudges. Data were analyzed using one-way analysis of variance (ANOVA) with Tukey *post hoc* tests in R 4.4.2.

**Results:**

Compliance, measured by completed mobile ecological prospective assessments (mEPAs), varied significantly across most phases. Comprehensive nudging (Phase 1) yielded the highest completion rates and fastest response times, which declined following the removal of personalized scientist-triggered nudges. Loss of personalized scientist-triggered nudges had the most substantial impact on compliance.

**Conclusions:**

Consistent system-triggered reminders and personalized “Best Picture” nudges were most effective in sustaining compliance. Findings highlight the importance of integrating personalized, two-way communication features into digital health platforms to strengthen engagement in rural Indigenous communities. By enabling real-time interaction between youth and scientists, the platform supported integration across health, education, and research sectors. Its human-controlled backend and customizable citizen-facing interface reflect principles of human-centered AI, emphasizing trust and autonomy. This approach offers a scalable model for ethical, effective digital interventions that balance technological precision and participant agency.

## Introduction

Health systems worldwide are struggling to meet evolving public health needs, such as managing chronic illnesses, addressing the growing youth mental health crisis, and responding effectively to public health emergencies such as pandemics (Khatri et al., 2023; Filip et al., 2022; Khetrapal & Bhatia, 2020; Kruk et al., 2018). These shortcomings stem from the limitations inherent in traditional health systems, which typically operate independently from other sectors, such as education and social services (Kruk et al., 2018; Institute of Medicine (US) Committee on the Health Professions Education Summit, Greiner & Knebel, 2003). In response to these limitations, a shift towards integrating systems (*e.g*., health, education, social services) is necessary to effectively deliver services (Komashie et al., 2021; McCullough, Leider & Phillips, 2020; Lai et al., 2017). However, the integration of systems in this digital age requires a citizen or patient-centered focus that can ethically leverage big data outside health systems to enhance overall quality of care (Katapally, 2020a). Such approaches which meaningfully engage or facilitate citizen participation—including digital citizen science—can lead to improved health outcomes, *i.e*., health systems transformation (Katapally, 2020a; Katapally & Bhawra, 2023; Buchan, Katapally & Bhawra, 2024).

Citizen science is an approach that offers health systems a shift in perspective by empowering patients to actively participate in healthcare delivery or interventions through collaboration with care providers and scientists (Katapally, 2020a, 2019). For instance, in mental healthcare delivery, involving patients as citizen scientists facilitates a deeper sense of ownership and collaboration, which is crucial in addressing mental health challenges (Todowede et al., 2023; Marks et al., 2022). To truly integrate citizen science in health systems transformation, digital health platforms designed with adaptive user interfaces could be utilized in healthcare interventions to drive communication between citizens and care providers through ubiquitous digital devices (*i.e*., smartphones) (Bhawra et al., 2022; Katapally & Chu, 2020). However, while citizen science has been applied in fields like environmental monitoring, there remains limited evidence on how it can be operationalized within digital mental health interventions to enable real-time participant feedback and influence engagement strategies. Digital health platforms are digital applications or technologies used to support health (*e.g*., virtual healthcare), often consisting of frontend (*i.e*., app interface) and backend (*i.e*., digital dashboards/analytics) systems (Bhawra et al., 2022; Katapally & Ibrahim, 2023). These platforms can be accessed by various stakeholder groups including citizens, care providers, and scientists on a range of digital devices to encompass electronic health (eHealth), mobile health (mHealth) and ubiquitous health (uHealth) (Government of Canada CI of HR, 2021; O’Hara & Toussaint, 2021; World Health Organization, 2019; Oh et al., 2015).

From a health interventions perspective, key challenges for sustaining long-term benefits are poor compliance (Brown, Hayashi & Eisenberg, 2019). This challenge is exacerbated when interventions are conducted outside of clinical settings, as this contributes to a disconnect between participants and care providers (Brown, Hayashi & Eisenberg, 2019; Doyle, Lennox & Bell, 2013). Factors such as competing priorities, convenience, and limited resources can further contribute to low adherence rates among participants (Chakrabarti, 2014). As a result, there is a need to enhance engagement strategies and address barriers to compliance to maximize the effectiveness and sustainability of health interventions. Digital health platforms have the potential to address these challenges when they are used to implement health interventions across systems by: (1) bridging the communication gap between care providers and citizens; (2) fostering ongoing engagement and improving adherence; and (3) providing real-time care to improve access as well as effectiveness of service delivery (Katapally, 2020b; Kashef, Visvizi & Troisi, 2021; Chen et al., 2022; Ranchordás, 2020).

Through human-computer interaction (HCI), defined as two-way information transfer between human- and computer-enabled systems (Kashef, Visvizi & Troisi, 2021), digital health platforms can revolutionize how citizens and care providers connect in real-time. This study focuses on mobile-based digital health platforms consisting of a citizen-facing app and a scientist-facing backend dashboard, designed specifically for mental health data collection, communication, and engagement. For instance, to encourage and support citizens in making informed decisions, a system of digital nudges or notifications rooted in HCI principles can be pushed to citizen-owned ubiquitous digital devices (Chen et al., 2022; Ranchordás, 2020; Leimstädtner, Sörries & Müller-Birn, 2023). Digital nudges refer to momentary reminders designed to understand and improve citizen decision-making processes (Peer et al., 2020). Grounded in behavioral economics, nudges leverage well-documented cognitive biases, such as default bias, social proof, and loss aversion, to influence behavior in subtle, non-coercive ways (Plak, van Klaveren & Cornelisz, 2023). Nudges can encompass various strategies, including automated and personalized reminders (Plak, van Klaveren & Cornelisz, 2023) aimed to gently guide participants towards making decisions without restricting their freedom of choice (Mertens et al., 2022).

While digital nudging has gained traction in behavioral health interventions, it is rarely integrated with digital citizen science approaches, which prioritize participant autonomy, co-creation, and active knowledge production (Katapally, 2020a, 2019; Marks et al., 2022). These two approaches, nudging and citizen science, are conceptually distinct and sometimes philosophically opposed. Nudging introduces behavioral influence, often subtly, while citizen science centers agency and voluntary participation. This tension is especially important to acknowledge in public health interventions involving marginalized communities, where trust, transparency, and self-determination are critical (Cappa et al., 2020; Schmidt & Engelen, 2020). However, we argue that these frameworks can be ethically and practically reconciled through human-centered digital design (Kang et al., 2025; Zikmund-Fisher et al., 2022). Rather than deploying nudges as a covert behavioral strategy, it can instead act as a transparent, co-designed feature of the digital platform to sustain involvement in co-owned initiatives. This integration of nudging within a citizen science framework preserves autonomy while offering practical solutions to real-world challenges of adherence and participation.

Despite increasing evidence supporting the effectiveness of digital nudging in various healthcare contexts, including electronic health record prompts in primary care (Nguyen et al., 2024), cancer screening behaviors (Wang et al., 2025), and digital transformation efforts in healthcare (Stoumpos, Kitsios & Talias, 2023), most of this research focuses on clinical or urban settings, often without grounding in human-centered design or participatory research frameworks. For instance, a recent mapping review found that while human-centered design is increasingly referenced in digital mental health interventions, few studies meaningfully integrate and rely on it for their design approaches (Vial, Boudhraâ & Dumont, 2022). Moreover, while behavioral strategies such as gamified nudging have shown promise in influencing mental health service uptake among young adults (Piao & Joo, 2022), few studies have explored how digital nudging can be ethically implemented in non-clinical, school-based interventions within rural Indigenous communities, especially through citizen science approaches. Thus, no evidence exists that depicts how digital health platforms can use varied HCI-based nudging protocols to improve implementation of youth mental health interventions by integrating multiple systems across health and education while centering youth as active participants in the research process through digital citizen science approaches.

To address this gap, this study utilized a mobile-based digital health platform that utilizes human-centred artificial intelligence (AI) to embed an HCI-based nudging system into an ongoing mental health intervention targeting Indigenous youth in remote Canadian communities. Human-centred AI is an approach that prioritizes the needs, values, and wellbeing of humans by actively engaging citizens. The platform included a youth-facing mobile app for data submission and engagement, and a scientist-facing dashboard for monitoring responses and sending nudges in real time. Using a natural experimental design, the study aimed to empirically evaluate how varying nudging protocols affected youth compliance—defined as the number of completed mEPA surveys and the time taken to respond to system-triggered prompts—over time, within a platform that integrates health and education systems through digital citizen science. Specifically, our study hypothesizes that there is a significant difference in youth compliance levels across different phases of nudging. To evaluate this, we used mobile ecological prospective assessments (mEPAs), an adaptation of ecological momentary assessments designed to capture real-world behaviors in a prospective, participant-driven manner. Using smartphone-based prompts, mEPAs allow youth to provide timely data about their daily experiences while reducing response burden and maintaining ecological validity. This hypothesis is grounded in prior literature, which suggests that consistent, personalized nudging improves adherence and accelerates response times in digital mental health interventions (Yeung et al., 2025).

## Materials and Methods

### Smart indigenous youth initiative

This study is part of the Smart Indigenous Youth initiative, a 5-year longitudinal community trial set in rural and remote regions of the Canadian prairie province of Saskatchewan. The trial embeds culturally appropriate land-based active living programs into school curricula to promote mental health and minimize substance abuse among Indigenous youth. These school-based programs were delivered during instructional hours, led or supervised by local educators, and tailored by each school to reflect their unique cultural and environmental contexts. While the broader goal of the initiative was to assess long-term mental health outcomes through sustained digital engagement, an unexpected disruption in the platform’s nudging system during the final year created a serendipitous natural experiment. This event allowed us to examine how variations in digital nudging affected youth compliance with the intervention over time.

The initiative was developed in collaboration with Indigenous communities and is grounded in a commitment to cultural safety and self-determination. In Canada, Indigenous Peoples (Walker et al., 2023) are among the most disadvantaged and vulnerable groups (Katapally, 2020b; United Nations, 2009) due to the legacy of colonization, intergenerational trauma, and continuing historical injustices (Katapally, 2020b; Bombay, Matheson & Anisman, 2014; Sherwood, 2015), which have contributed to systemic barriers in accessing culturally appropriate mental health services. These challenges often result in reduced engagement with conventional care models among Indigenous youth (Subica & Link, 2022; Smye et al., 2023). By centering local knowledge systems and prioritizing community-led design, the Smart Indigenous Youth initiative aims to rebuild trust, enhance youth participation, and support improved mental health outcomes through culturally grounded interventions.

**Figure 1:**
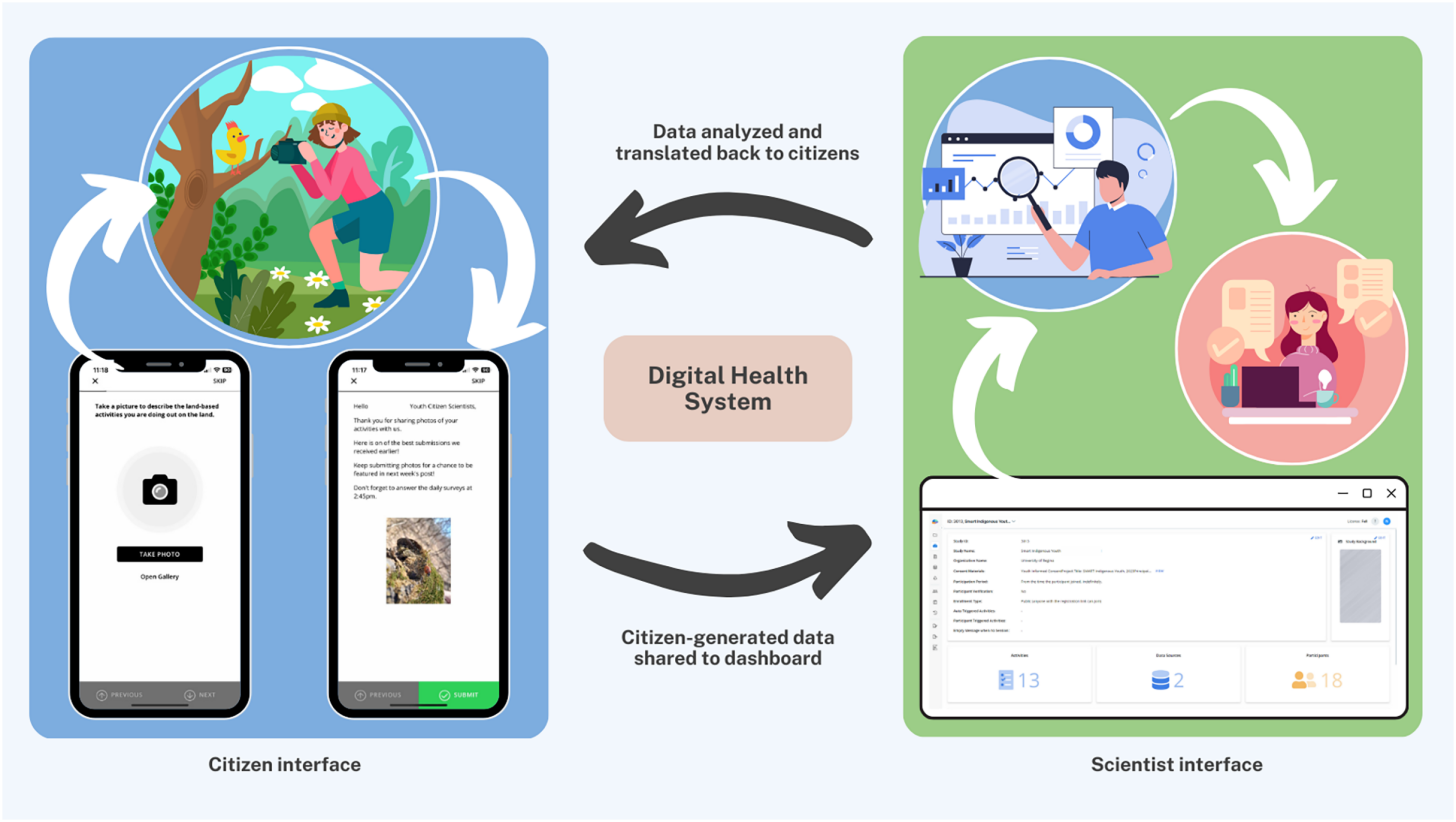
The digital health platform: citizen interface + scientist interface.

The Smart Indigenous Youth initiative is implemented by integrating services supported by the Saskatchewan ministries of health, education, and sport (Katapally, 2020b). Central to this integration is the use of interactive technologies, including a digital health platform designed with adaptive user interfaces, which foster real-time communication between citizens and scientists. The primary approach in implementing the initiative is digital citizen science, which in this context refers to Indigenous youth participating as active contributors and co-designers in a health intervention by sharing data and receiving feedback through a digital platform. This human-centred AI platform consists of two interconnected components: a youth-facing mobile app and a scientist-facing dashboard. The mobile app enables youth to submit data and receive various nudges (automated and personalized), while the dashboard allows academic scientists to visualize real-time data, monitor youth engagement, and trigger nudges accordingly. Together, these interfaces facilitate remote, real-time, bidirectional communication and shared decision-making between youth citizen scientists and academic researchers (Fig. 1).

In this study, all youth contributed as citizen scientists by engaging with the research team at the beginning, during, and end of each school term (Buchan, Katapally & Bhawra, 2024). Youth citizen scientists facilitated both the evaluation of their school’s land-based intervention and the study design itself to cocreate knowledge as equal partners (Katapally, 2020b). They played an important role in shaping data collection by communicating with the academic scientists in real-time *via* the app to provide feedback on data collection strategies. The citizen scientists played a key role in determining study start date and the app’ adaptive interface enabled youth to customize nudging notification times (*e.g*., morning, afternoon, evening) to enhance engagement through personalized digital interactions.

As part of this initiative, in partnership with the research team, the four participating schools located in rural and remote Indigenous communities developed their own educational component consisting of land-based active living programs that were specific to their culture, community, geography, and language. Each school collaborated with local Elders, Knowledge Keepers, and community leaders to identify activities that reflected regional traditions and environmental conditions. Land-based activities included traditional hunting, trapping, fishing, foraging, plant identification, as well as recreational activities such as canoeing and hiking. Implementation occurred collaboratively between school personnel and youth, ensuring cultural appropriateness and local relevance. In essence, although the overall implementation structure was identical, including implementation in school settings, during school hours, and guided by educators and community leaders, each school selected and implemented their own intervention. The basic structure of the intervention relied on the school terms (intervention periods/units), which also influenced the land-based activities that depended on seasonality.

As described earlier, youth used the app to complete daily mEPA surveys throughout the intervention period. mEPAs allow participants to provide data about their behaviors and perceptions through their mobile devices, reducing response burden while preserving ecological validity (Ibrahim, Hammami & Katapally, 2023). For instance, mEPAs were used to measure changes in physical activity on a daily basis, where youth were able to report at the end of the day prospectively rather than every time they moved (Ibrahim, Hammami & Katapally, 2023). Prospective ecological assessments assess participants’ experience/behaviors in real-time, and in the real-world, where researchers use sampling and monitoring strategies to assess phenomena as they occur in natural settings (Ibrahim, Hammami & Katapally, 2023; Shiffman, Stone & Hufford, 2008). These measures captured physical activity, sedentary behavior, mental health, and substance abuse, among other behaviors and outcomes (Katapally, 2020b). Thereafter, each school initiated their land-based active living programs. To understand the impact of these programs on youth mental health during intervention period, youth citizen scientists were engaged in real time *via* their own smartphones to identify the impact of land-based activities on their mental health. To ensure consistent engagement with the research team (*i.e*., academic scientists), a series of automated system-triggered, as well as non-personalized and personalized scientist-triggered nudges were deployed throughout the intervention period.

**Figure 2:**
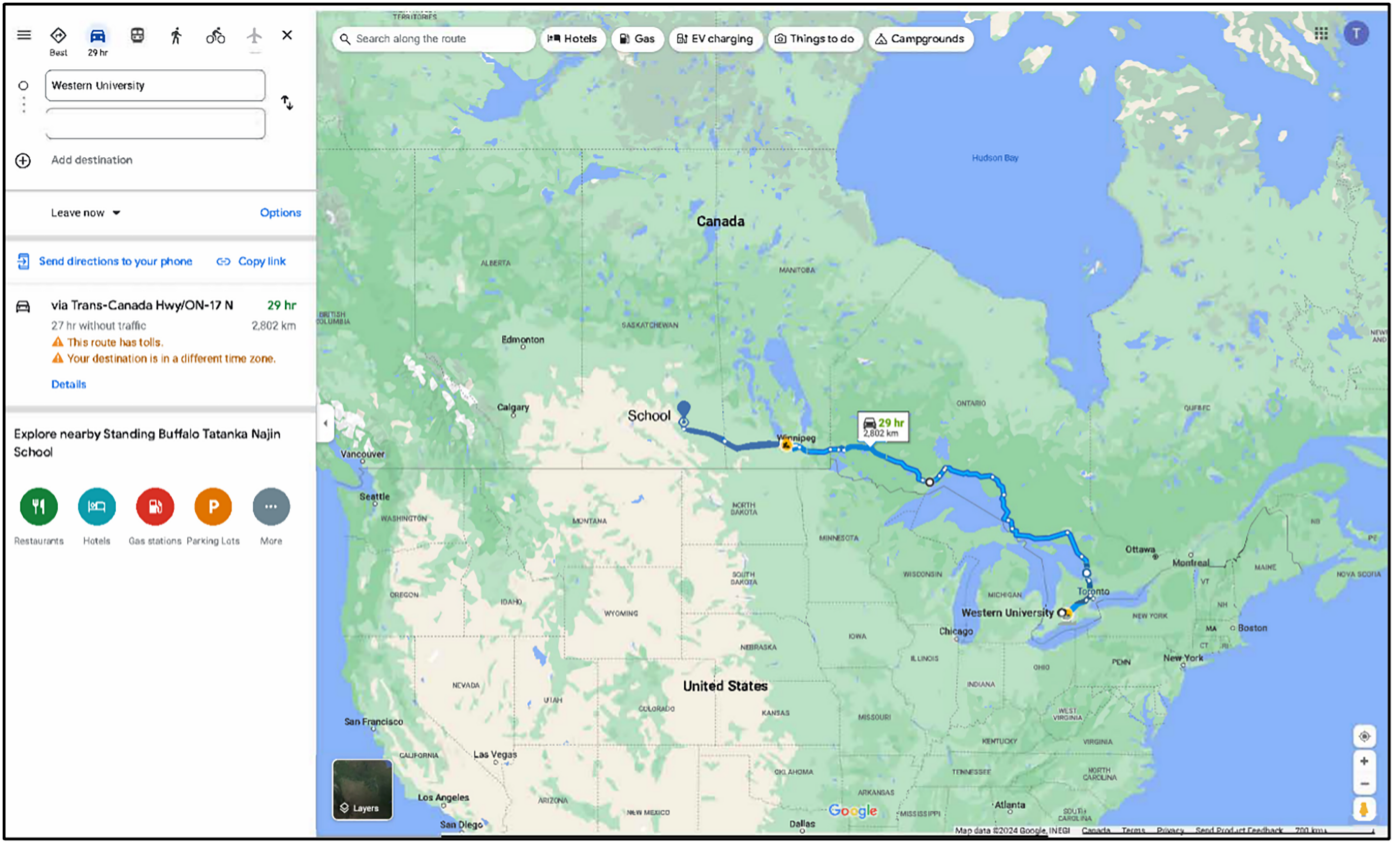
Google map showing distance between the participating school and the research team. Map data ©2024 Google, INEGI.

This study focuses specifically on the impact of these nudges on youth compliance to the intervention in one of the schools that participated in the Smart Indigenous Youth initiative in the winter of 2023. Figure 2 (Google Inc., Mountain View, CA, USA) shows the distance between the participating school and the research team. Ethics approval for the study was obtained through a synchronized review protocol coordinated between the Research Ethics Boards of the University of Regina and the University of Saskatchewan (REB # 2017–29). While these institutions do not provide a public lookup system for external verification of ethics protocols, we confirm that full ethical approval was granted prior to data collection and that all study procedures adhered to the approved protocol. Parents/guardians were required to provide implied informed consent for their children’s participation. Participation in the study was voluntary, and all youth citizen scientists provided informed consent *via* the smartphone app before joining the study.

### Study design: embedding nudging protocols with integrated knowledge translation

This quasi-experimental study was conducted in February 2023 to evaluate a serendipitous and unplanned natural experiment as part of the larger Smart Indigenous Youth initiative. Natural experiments are real-world events that are beyond the control of scientists (Craig et al., 2017). The participating school initiated a 4-month land-based active living program that was specific to their culture, community, geography, and language. During the intervention period from February 16^th^ to June 25^th^, 2023, youth engaged with the research team in real-time *via* the smartphone app. To ensure consistent youth engagement in the trial, a comprehensive nudging system built upon HCI principles was developed, including user-centered design, transparency, and feedback. User-centered design allowed participants to customize the timing of nudges to suit their schedules, transparency was achieved by informing participants of the data being collected and how it was used to shape the intervention, and feedback was embedded through a real-time dashboard that allowed scientists to monitor engagement and respond with tailored nudges, while youth received visual and interactive feedback (*i.e*., being featured in the “Best Picture” message) to reinforce participation. In the context of this study, system-triggered nudges are defined as automated notifications triggered by the digital health platform on a fixed schedule *i.e*., time-triggered. For example, nudges that are automatically sent to participants to serve as reminders to complete surveys. Alternatively, scientist-triggered nudges include notifications administered by the research team using the backend dashboard.

This particular natural experiment captures how engagement patterns shift when nudging systems are unintentionally disrupted in an uncontrolled, real-world setting. Unlike controlled trials, this setting offers insights into the durability and fragility of digital engagement strategies, allowing us to examine which nudging components are essential for sustaining youth participation. It also provides a rare opportunity to observe how different types of nudges (*i.e*., automated, non-personalized, and personalized) function in isolation and in combination, revealing their relative effectiveness in promoting compliance in a school-based mental health intervention.

**Figure 3:**
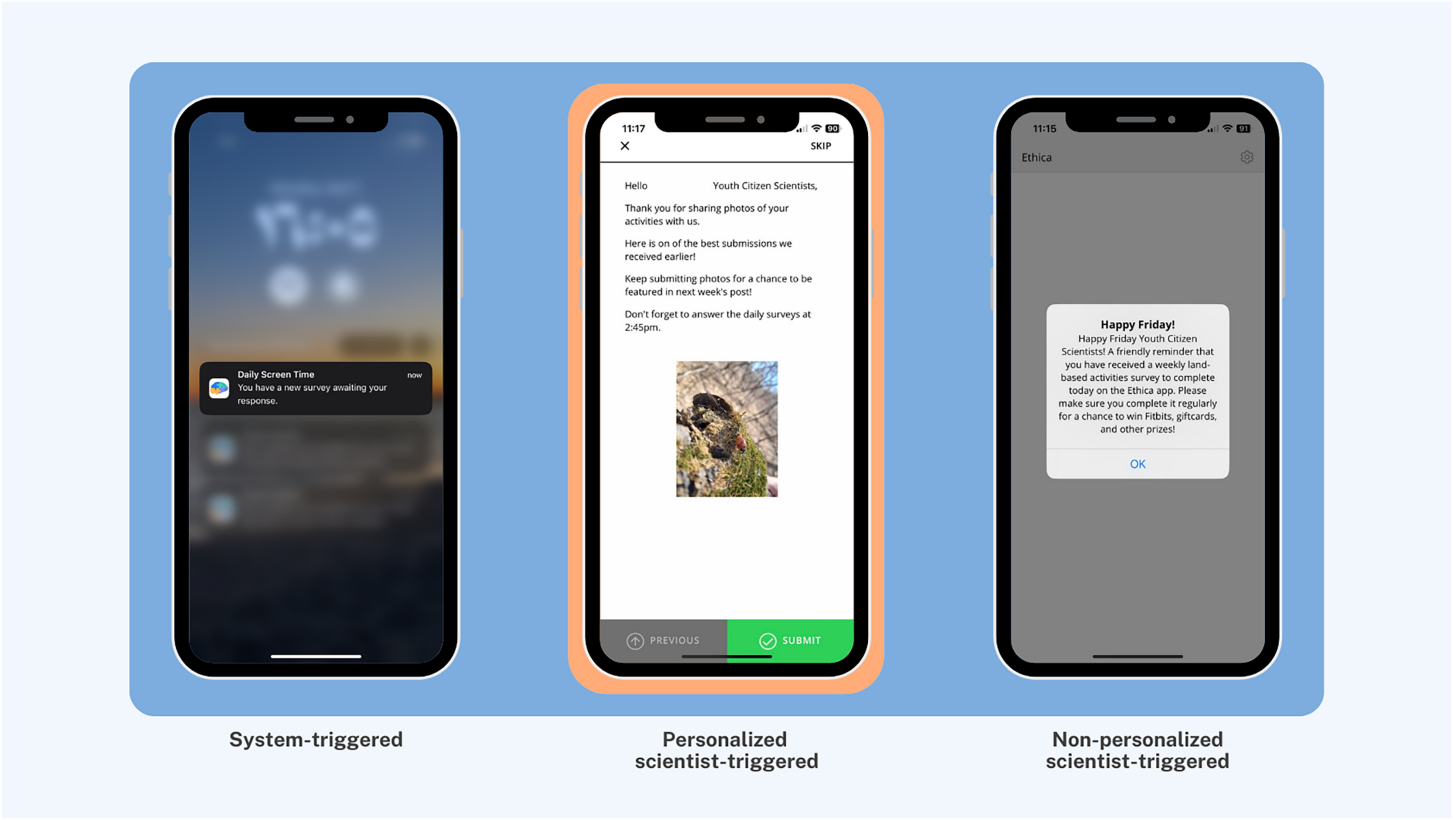
Citizen interface with examples of system-triggered, personalized scientist-triggered, and non-personalized scientist-triggered nudges.

**Figure 4:**
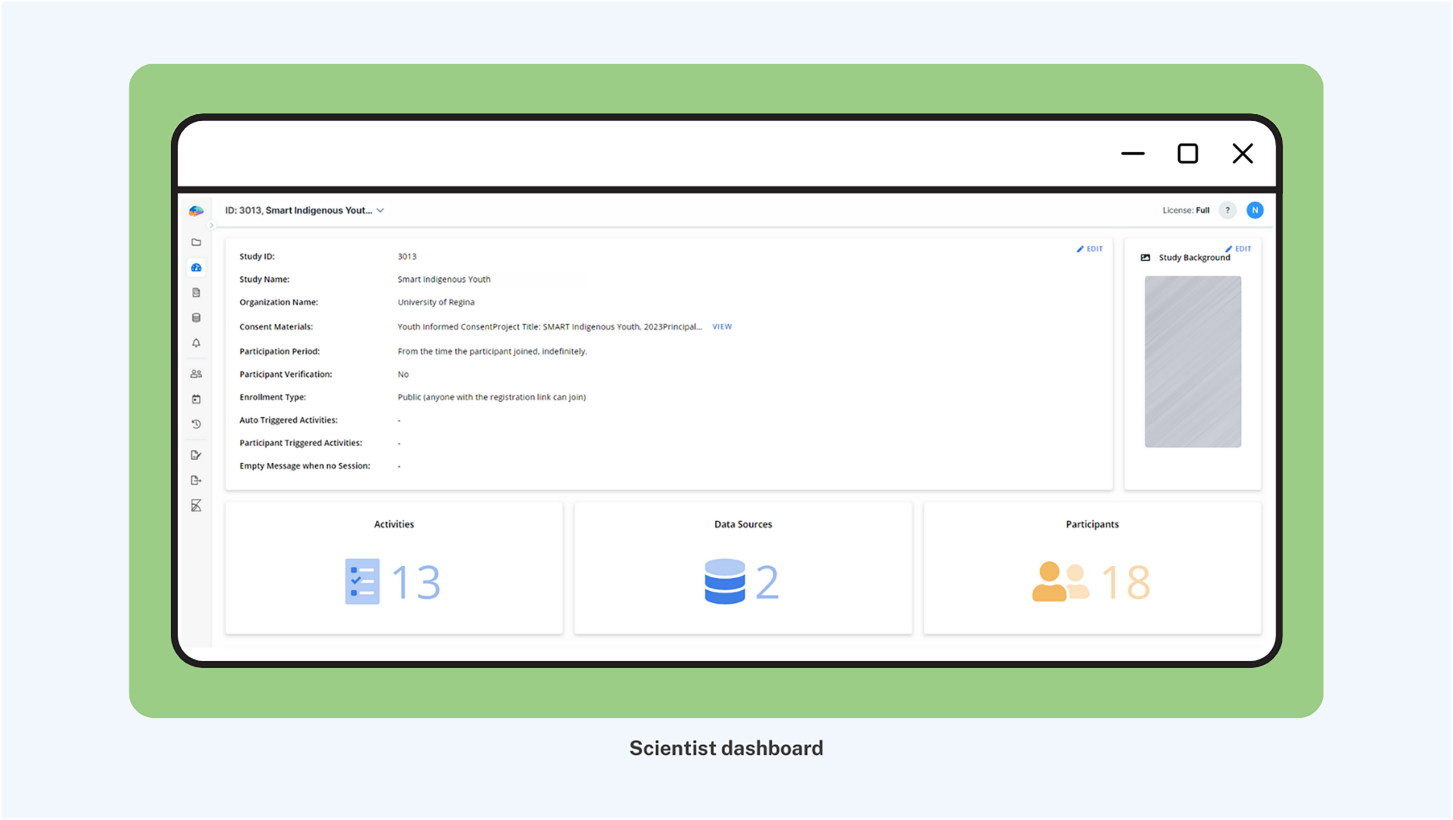
Scientist interface of backend dashboard with citizen-generated data.

More importantly, to maximize participant compliance, nudging was embedded with integrated knowledge translation, an approach in health systems research for actively involving knowledge users (*i.e*., patients, clinicians, policymakers) in the research process from design through dissemination (Lawrence, Bishop & Curran, 2019). Nudges were categorized into three distinct types of smartphone app notifications to facilitate youth engagement and maximize interactivity (Fig. 3): (1) System-triggered notifications were automatically triggered daily asking youth to complete mEPAs capturing their physical activity and sedentary behavior. These nudges supported continuous data flow from participants to scientists, enabling real-time insight into youth behaviors that could later be shared back in aggregated form, fostering reciprocal knowledge exchange. (2) Non-personalized scientist-triggered notifications triggered every Friday asking youth to provide input about the impact of land-based activities on their mental health (*i.e*., the intervention). These nudges acted as regular check-ins, reinforcing bidirectional communication and affirming participants’ role as co-contributors to the intervention. (3) Personalized scientist-triggered notifications titled “Best Picture” were triggered every Monday to showcase the best images submitted by youth from the previous week, showing their participation in the land-based activities (*i.e*., the intervention). These nudges fed citizen-generated content back to the group in a curated, culturally resonant format that promoted social recognition, increased transparency, and reinforced participation through positive feedback loops. By sharing citizen-generated data from the previous week, specifically images sent by youth, these weekly notifications were personalized to reflect the cohort’s evolving participation in the study. These nudges were administered using the backend dashboard by the research team, which provided access to study activities, participation levels, and citizen-generated data (Fig. 4).

**Figure 5:**
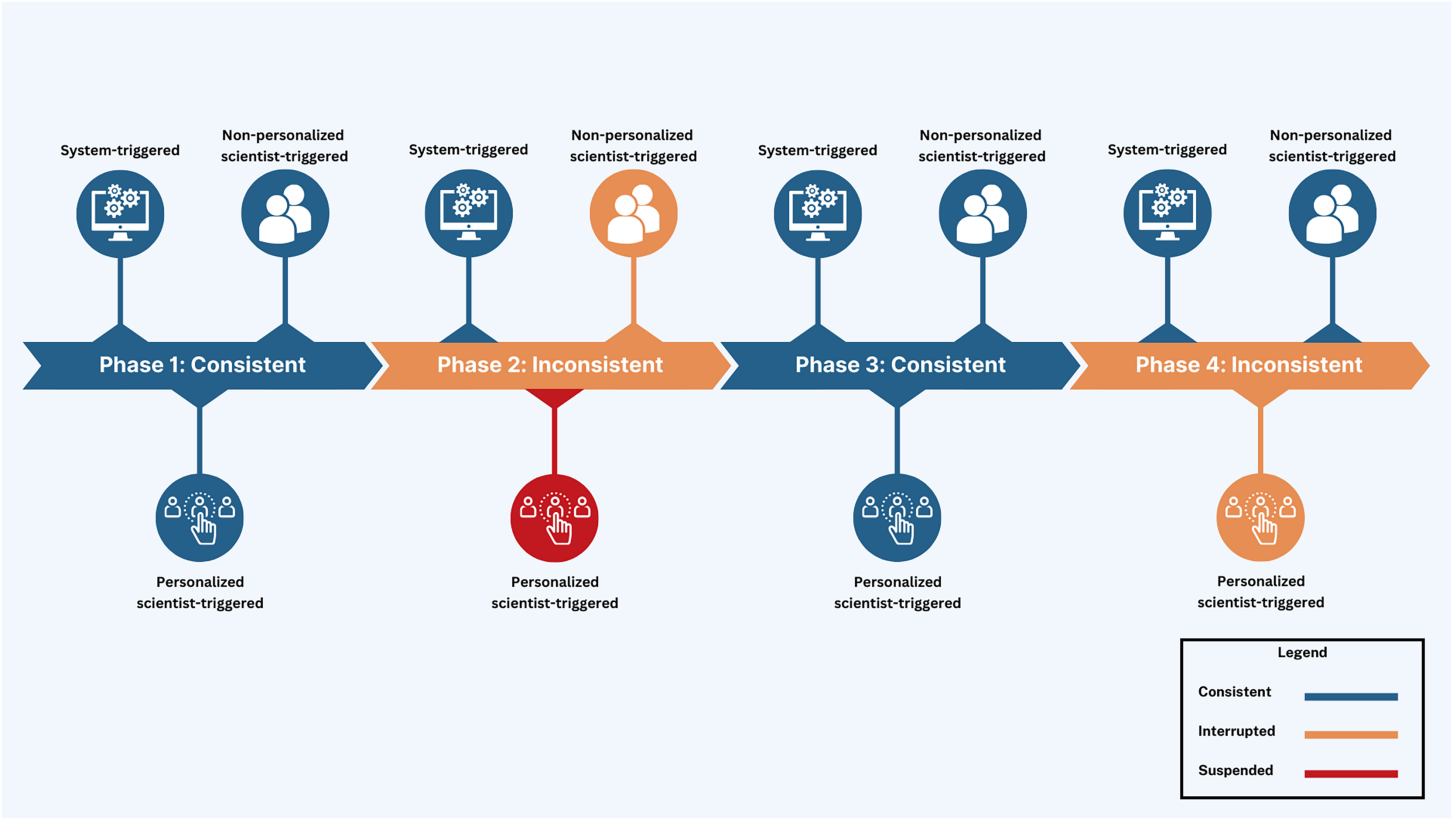
Visual depiction of study phases with deployed nudge types.

The nudging design was initially set up as a continuous digital engagement protocol throughout the intervention period. However, an unplanned interruption of non-personalized scientist-triggered and personalized scientist-triggered notifications due to cloud computing system errors transformed the study into a serendipitous natural experiment, *i.e*., an unintentional change in the nudging protocol. This altered the design into four intervention phases: Phase 1 (Feb 21–Mar 26, 2023; 5 weeks): consistent nudging with all nudge types—system-triggered, non-personalized scientist-triggered, and personalized “Best Picture” nudges, Phase 2 (Mar 27–Apr 23, 2023; 4 weeks): interruption of non-personalized and suspension of personalized scientist-triggered nudges with the continuation of system-triggered nudges, Phase 3 (Apr 24–May 21, 2023; 4 weeks): reintroduction of consistent nudging using all nudge types, and Phase 4 (May 22–Jun 25, 2023; 5 weeks): interruption of personalized scientist-triggered nudges with the continuation of system-triggered and non-personalized nudges (Tables 1 and 2, and Fig. 5). This unintentional phasing provided an opportunity to assess how varying levels of interactivity within the digital platform influenced user compliance. Each phase began on Mondays with the administration of personalized scientist-triggered nudges and concluded on Sundays. This structure was designed to align with the land-based activities conducted by the school each week.

**Table 1:** Intervention period, definition, and duration.

Intervention period	Definition	Duration
Phase 1	Consistent nudging using all nudge types (system-triggered + non-personalized scientist-triggered + personalized scientist-triggered)	Feb 21^st^–March 26^th^, 2023 (5 weeks)
Phase 2	Interruption of non-personalized scientist-triggered nudges and suspension of personalized scientist-triggered nudges (system-triggered + inconsistent non-personalized scientist-triggered only)	March 27^th^–April 23^rd^, 2023 (4 weeks)
Phase 3 (Phase 1 repeated)	Reintroduction of consistent nudging using all nudge types (system-triggered + non-personalized scientist-triggered + personalized scientist-triggered)	April 24^th^–May 21^st^, 2023 (4 weeks)
Phase 4	Interruption in personalized scientist-triggered nudges (system-triggered + non-personalized scientist-triggered + inconsistent personalized scientist-triggered nudges)	May 22^nd^–June 25^th^, 2023 (5 weeks)

**Table 2:** Number of nudges triggered in each study phase.

Intervention period	Duration (weeks)	Number of system-triggered nudges released	Number of non-personalized scientist-triggered nudges released	Number of personalized scientist-triggered nudges released
Phase 1	5	34	5	5
Phase 2	4	28	1	0
Phase 3	4	28	4	4
Phase 4	5	28	4	1

### Data collection procedures and study measures

**Figure 6:**
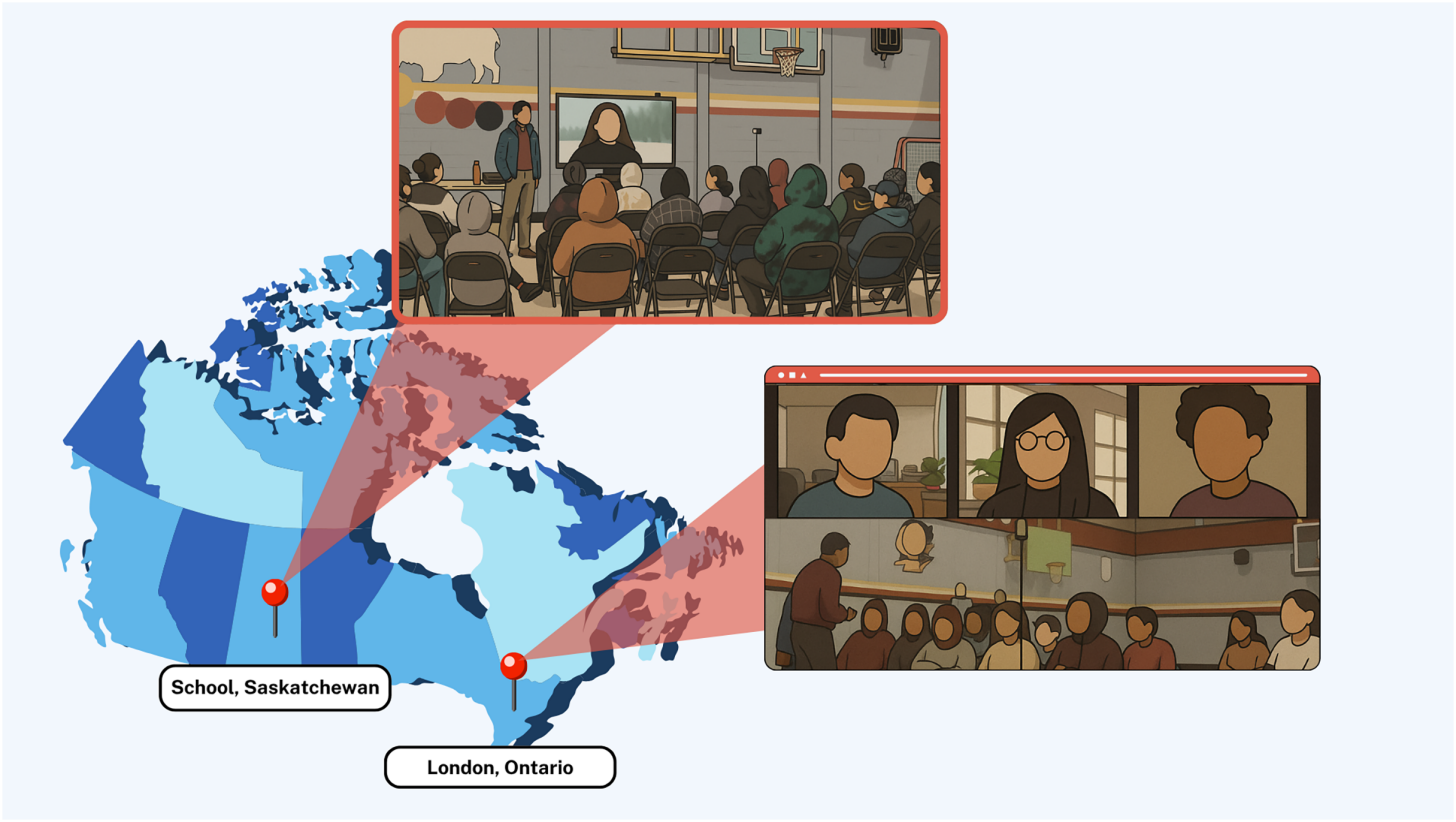
Hybrid (in-person + remote) engagement session on day 1 of the study.

The youth participated in an initial hybrid (in-person + remote) engagement session on day 1 coordinated by the research team and the school administration, lasting for a total of 90 min including instructions and app setup (Fig. 6). Three members of the research team travelled to the school to host the engagement session, while the rest of the research team joined remotely to provide support. During this engagement, the research team welcomed only those youth whose parents had provided consent and explained their role and contributions. The research team conducted a presentation to describe the study and demonstrated how to use the custom-built app, which included demonstrations of mEPAs among other tasks. The research team also answered queries and concerns of youth, and assisted youth in downloading the app onto their respective smartphones.

To join the study, youth downloaded the app from either the App Store or Google Play Store. After installing the app and before participating in any data collection, informed consent was obtained digitally *via* the app. This process included a detailed explanation of the study’s purpose, procedures, potential risks and benefits, data privacy protections, and participant rights (including the right to withdraw at any time). Once consent was provided, the land-based intervention began, and youth were remotely engaged by the scientists throughout the study period. Data collection consisted of system-triggered surveys, and system- and citizen-triggered mEPAs to capture a range of health behaviors and outcomes, and more importantly, youth perceptions of the impact of the intervention on their mental health (Ibrahim, Hammami & Katapally, 2023). At the initiation of our study, youth completed a series of one-time baseline validated surveys. Demographic and general wellbeing data were collected to contextualize youth characteristics and assess potential influences on digital engagement with the platform. Measures related to bullying, daily activities, school environment, and family and peer support were included due to their well-established associations with mental health outcomes and patterns of intervention compliance. Additional variables such as mental health and substance use, land-based activities, physical activity, and sitting and screen time were collected to capture a holistic understanding of youth health behaviors and their relevance to the aims of the intervention. Thereafter, a series of mEPAs were deployed longitudinally throughout the study period to capture youth perceptions of the intervention as well as change in behaviors and outcomes (Table 3). These mEPAs were released during school hours to ensure internet access for all youth involved in the study. Every Friday, a weekly mEPA was triggered to capture youth perceptions of the intervention (land-based activities) as well as its impact on their mental health. Moreover, youth had the freedom to trigger a “Take a Picture” mEPA anytime to capture land-based activities they did as part of the intervention, which enabled participants to prospectively capture images and provide descriptions of the land-based activities. Physical activity, as well as screen time and sedentary behavior were prospectively assessed using daily time-triggered mEPAs. Compliance consisted of (1) the total number of responses received from daily physical activity, and screen time and sedentary behavior mEPAs per day; and (2) the time taken to respond to daily physical activity, and screen time and sedentary behavior mEPAs per day. Response time was derived by calculating the difference between the nudge time—the timestamp when the system-triggered mEPA was delivered to the participant’s device—and the timestamp of the participant’s completed submission. While no minimum response thresholds were required for study participation, analyses focused on mEPAs submitted within 24 h of delivery to reflect timely engagement and practical compliance with the intervention.

**Table 3:** Study measure, type, name, logic, and frequency.

Ecological measures	Description of data	Triggering logic	Trigger time	Triggering frequency
Baseline survey	Demographics, social and contextual factors, health behaviors and outcomes	Time-triggered	At study registration on Day 1	One-time
Land-based activities mEPA	Perception of intervention and impact on youth health	Time-triggered	2:45 pm	Weekly
Physical activity mEPA	Daily physical activity	Time-triggered	2:45 pm	Daily
Screen time and sedentary behavior mEPA	Daily screen time across different devices and sedentary behavior	Time-triggered	2:45 pm	Daily
Take a picture mEPA	Perception of intervention	Citizen-triggered	N/A*	Anytime

**Note:**

mEPA, mobile ecological prospective assessment. *N/A, trigger time does not apply because youth can launch the mEPA anytime they wish.

### Data and risk management

The app used throughout the study was built to ensure confidentiality, data safety, and security (Katapally et al., 2018). Specifically, mEPA responses, user identifiers, and associated timestamps were encrypted both at rest and during transmission to a secure cloud server. The custom-built smartphone app was also restricted from accessing personally identifiable information present in youth smartphones (*e.g*., site visited, location, and contact list).

During in-person recruitment sessions, the risk and privacy management options were made clear to participants through the digital informed consent process. These included the potential for data confidentiality breaches, which were mitigated by assigning anonymous identifiers and securing all responses *via* encryption. Participants were also assured that their involvement was entirely voluntary and that non-participation would not affect their standing in the school or the community. Within the app, youth were given the option to drop out or pause data collection anytime during the study period. Also, participants could decide to upload their data only when they had access to Wi-Fi or when their smartphones were plugged into a power source. Clear instructions were provided within the app for participants who wished to withdraw from the study at any point in time (Ibrahim, Hammami & Katapally, 2023; Katapally et al., 2018).

### Data analyses

The primary purpose of our data analyses was to ascertain if a significant difference exists across the four study phases in two compliance indicators: (1) mEPA responses, and (2) the time taken to respond to time-triggered mEPAs. An mEPA response was defined as the successful and complete submission of an mEPA questionnaire (either time- or citizen-triggered) by a participant through the smartphone app. Partially completed or abandoned questionnaires were not counted. Response time was calculated as the elapsed time (in minutes) between the moment a system-triggered mEPA notification was sent to the participant and the moment a fully completed response was submitted.

Data analyses were conducted in R, an open-source and free statistical software program (Paradis, 2005). Frequencies and percentages were used to present the distribution of participants’ socio-demographic characteristics. Descriptive analyses, including mean and standard deviation, were conducted to analyze the spread and variations of mEPAs across the four phases of both time- and citizen-triggered mEPAs. Finally, one-way analysis of variance (ANOVA) was used to assess the overall differences in total mEPA responses and the time taken to respond to time-triggered mEPAs across the study phases. Since the study was carried out to compare the responses collected at each phase, the one-way ANOVA was appropriate to use. Additionally, the Tukey *post hoc* test was conducted for pairwise comparison between study phases (*i.e*., Phase 1 *vs* Phase 2; Phase 1 *vs* Phase 3; Phase 1 *vs* Phase 4; Phase 2 *vs* Phase 3; Phase 2 *vs* Phase 4; and Phase 3 *vs* Phase 4)—specifically, differences in mEPA responses, and time taken to respond to time-triggered mEPAs. The Tukey *post hoc* test was adopted to avoid multiple pairwise comparisons, which may lead to a type I error. All statistical significance were considered at *p* < 0.05.

## Results

### Sample summary

Sample descriptive statistics of youth participants (*n* = 29) are summarized in Table 4. The age range of youth spanned from 12 to 21 years, with the mean age being 13.68. The gender distribution among youth was predominantly male (*n* = 12, 57.14%), followed by female (*n* = 8, 38.10%), with a small percentage identifying as other (*n* = 1, 4.76%). In terms of ethnic identity, youth self-identified as mixed (*n* = 9, 42.86%), Dakota (*n* = 6, 28.58%), First Nations (*n* = 2, 9.52%), Cree (*n* = 2, 9.52%), Métis (*n* = 1, 4.76%), or Canadian (*n* = 1, 4.76%).

**Table 4:** Summary statistics for youth citizen scientists participating in this study (*n* = 29).

**Variables** *	**Mean (S.D.)**
mEPA responses (response/day)	3.25 (2.89)
Time taken to respond to mEPAs (min/day)	233.55 (216.11)
Age	13.68 (1.89)
**Age (years)**	**Frequency (%)**
12–15	20 (90.90)
16–18	1 (4.55)
19–21	1 (4.55)
**Gender**	
Male	12 (57.14)
Female	8 (38.10)
Other	1 (4.76)
**Parental education**	
Elementary school (some or completed)	4 (20)
Completed secondary/high school	2 (10)
Some post-secondary (University or college)	2 (10)
Received University or college degree/diploma	1 (5)
Do not know/Does not apply	11(55)
**Ethnicity**	
Mixed	9 (42.86)
First nations	2 (9.52)
Metis	1 (4.76)
Canadian	1 (4.76)
Dakota	6 (28.58)
Cree	2 (9.52)

**Note:**

* Reporting varied across demographic indicators.

In Phase 1, consistent nudging sustained high engagement, with participants completing an average of 3.06 responses per day and responding within 117.3 min on average. When nudging was disrupted in Phase 2 (non-personalized nudges only), engagement dropped to 1.31 responses per day and the response time lengthened to 168.0 min. Reintroducing the original protocol in Phase 3 modestly improved participation (1.50 responses per day; 179.2 min). However, Phase 4, which suspended personalized nudges entirely, yielded the lowest engagement, with only 1.08 responses per day and the slowest response time (255.5 min).

### Phase 1 (Five weeks: February 21^st^–March 26^th^, 2023)

**Figure 7:**
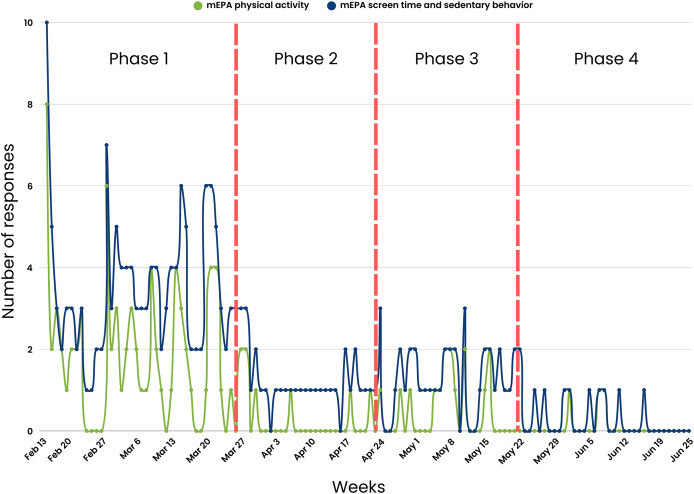
Time-triggered mEPA (physical activity and screen time and sedentary behavior) response rate across study phases.

**Figure 8:**
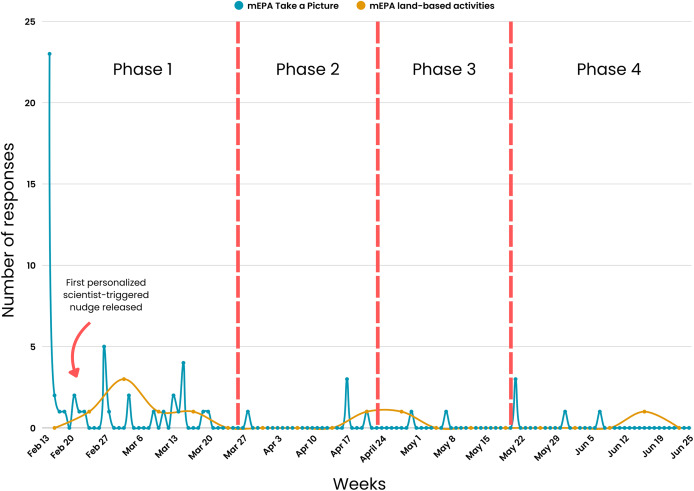
Citizen-triggered “Take a Picture” mEPA and weekly time-triggered land-based activities mEPA response rate across study phases.

**Figure 9:**
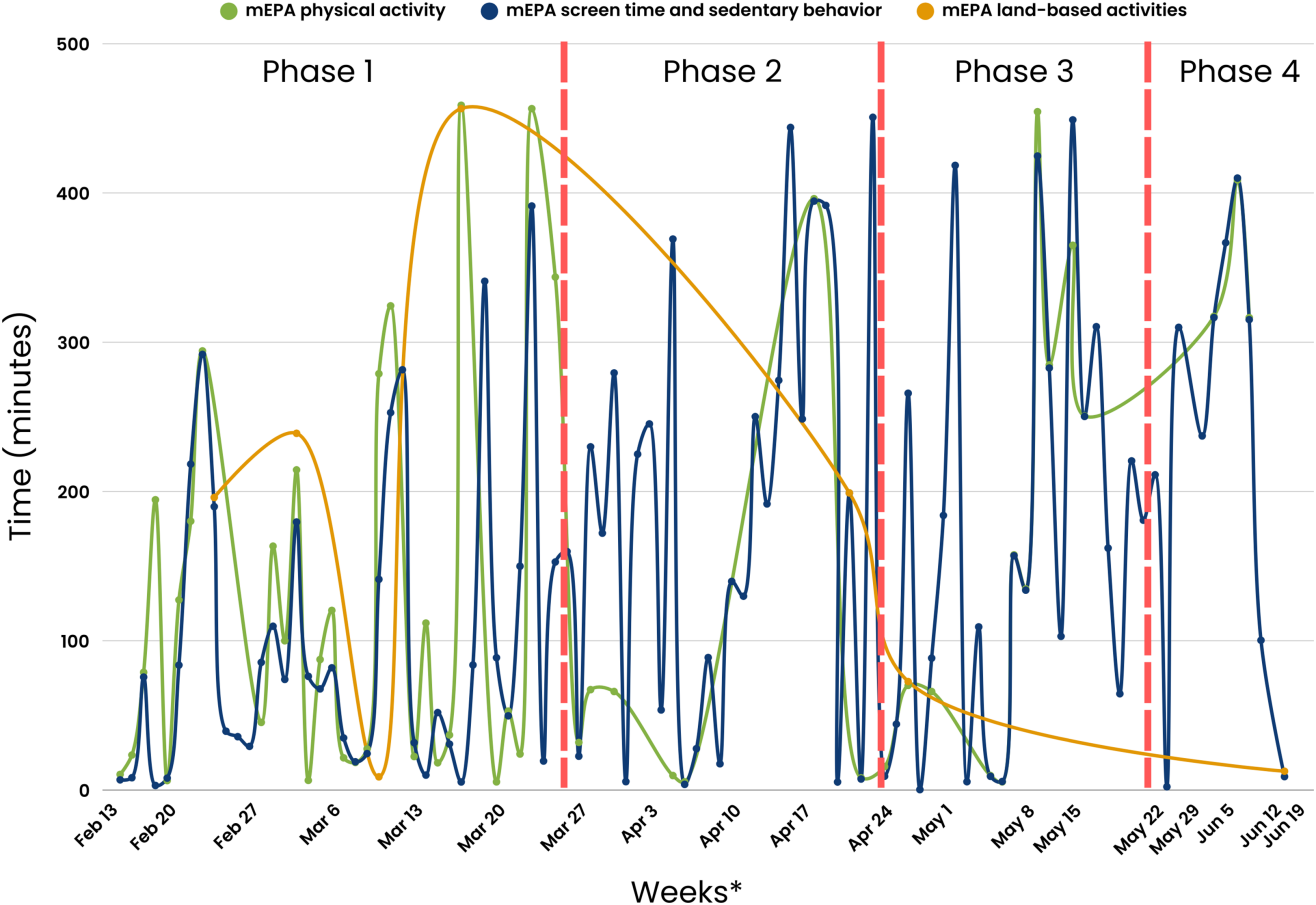
Average time taken to respond to time-triggered mEPAs (physical activity, screen time and sedentary behavior, and land-based activities) in minutes across study phases. *x-scale (Weeks) corresponds to the last day a response was received from participants.

A consistent nudging process was maintained weekly over the first 5 weeks of the study. The nudging process in Phase 1 included (1) daily system-triggered nudges reminding youth to complete daily physical activity and screen time and sedentary behavior mEPAs, (2) weekly system-triggered nudges prompting youth to complete the land-based activities survey every Friday, and (3) weekly personalized scientist-triggered nudges featuring participants’ best images from the previous week, released every Monday. The number of youth responses were at their highest during this phase across different measures, with physical activity mEPA responses at *n* = 72 (Fig. 7), screen time and sedentary behavior mEPA responses at *n* = 139 (Fig. 7), “Take a Picture” (of land-based activities) responses at *n* = 52 (Fig. 8), and land-based activities mEPA responses at *n* = 6. Additionally, the average minutes taken to respond to time-triggered mEPAs were at their lowest during Phase 1, with physical activity, and screen time and sedentary behavior mEPAs being answered in 137.17 (sd = 136.76) and 102.14 (sd = 100.11) min, respectively (Fig. 9). Conversely, the mEPA deployed weekly had the most delayed response time during Phase 1 at 229.71 (sd = 164.37) min.

### Phase 2 (Four weeks: March 27^th^–April 23^rd^, 2023)

After the fifth week of the study, the implementation of non-personalized scientist-triggered nudges and personalized scientist-triggered were temporarily interrupted due to a cloud computing system error, creating Phase 2 of the study. This suspension continued for four consecutive weeks. A notable decline in various metrics was observed during Phase 2 across different measures, with physical activity mEPA responses at *n* = 8, screen time and sedentary behavior mEPA responses at *n* = 34, “Take a Picture” (of land-based activities) responses at *n* = 5, and land-based activities mEPA responses at *n* = 1. Although, the average time taken to respond to daily time-triggered physical activity mEPA (84.75 min; sd = 154.61) and weekly land-based mEPA (199.09, sd = NA) min decreased in Phase 2, the time taken to respond to screen time and sedentary behavior mEPA increased (187.24, sd = 147.66) min. More importantly, after a sustained period of limited non-personalized scientist-triggered nudges and a lack of personalized scientist-triggered nudges, no responses were recorded in two of the three mEPAs (physical activity and “Take a Picture”, not screen time and sedentary behavior).

### Phase 3 (Four weeks: April 24^th^–May 21^st^, 2023)

The original consistent nudging protocol, which included non-personalized scientist-triggered nudges, personalized scientist-triggered nudges, as well as system-triggered nudges, was reintroduced in the tenth week of the study after the cloud computing system error was fixed. This initiated the next period of the study—Phase 3, which spread across 4 weeks. An increase in youth responses was observed in Phase 3 across most mEPAs, where physical activity mEPA responses were at *n* = 15, an 87.5% increase sedentary behavior and screentime mEPA responses were at *n* = 36, a 5.88% increase and “Take a Picture” mEPA at *n* = 6, a 20% increase. Land-based activities mEPA responses remained unchanged from Phase 2 at *n* = 1. Nevertheless, none of the responses returned to original levels observed during Phase 1 when the same consistent system-triggered and scientist-triggered nudging system was maintained. The average time taken to respond to time-triggered mEPAs varied from Phase 2 to Phase 3, with time taken to respond to physical activity mEPA increasing to 164.94 (sd = 153.96) min, a 94.62% increase and screen time and sedentary behavior mEPA, as well as weekly land-based activities mEPA decreasing to 185.93 (sd = 1,148.49), a 0.69% decrease, and 72.72 (sd = N/A) min, a 63.47% decrease, respectively.

### Phase 4 (Five weeks: May 22^nd^–June 25^th^, 2023)

Following the thirteenth week of the study, the reoccurrence of the same cloud computing system error reintroduced inconsistencies in personalized scientist-triggered nudges, resulting in the final period of the study—Phase 4. For the final 5 weeks of the study, there was a decrease in youth responses for most mEPAs including physical activity at *n* = 3, a 62.5% decrease, sedentary and screen time behavior at *n* = 11, a 66.64% decrease, and “Take a Picture” mEPA at *n* = 2, a 60% decrease, while land-based activities mEPA continued to remain unchanged at *n* = 1 across Phases 2, 3, and 4. The average time taken to respond to time-triggered physical activity and sedentary and screen time mEPAs continued to increase from Phase 2 to 347.52 (sd = 52.99) min, a 310% increase and 227.88 (sd = 145.33) min, a 21.70% increase, respectively. Similarly, the average time taken to respond to weekly land-based activities mEPAs decreased to its lowest at 12.63 (sd = N/A) min in Phase 4, in contrast to the increasing trend observed in other mEPAs.

### Citizen scientist compliance across the three phases of the natural experiment

Table 5 presents the ANOVA and independent the Tukey *post hoc* test. Phase 1 shows the highest average mEPA (response/day) (3.06, sd = 1.81), followed by Phase 2 (1.31, sd = 0.59), Phase 3 (1.50, sd = 0.56) and Phase 4 (1.08, sd = 0.28). The average time taken to respond to mEPAs (min/day) was: Phase 1—117.37; sd = 117.83, Phase 2—168.03; sd = 150.40, Phase 3—179.15; sd = 148.27, and Phase 4—255.49; sd = 138.07. The ANOVA results of the average mEPA responses (F(3,144) = 21.37, *p* < 0.001) and the time taken to respond to mEPAs (F(3,144) = 4.69, *p* = 0.003) indicate a significant difference across the four phases of this study. The mEPA responses in Phase 1 were significantly greater than Phase 2 as indicated by the positive difference. Additionally, the time taken to respond to mEPAs is lower in Phase 1 than Phase 2 as indicated by the negative difference.

**Table 5:** Overall mEPA responses and time taken to respond to time-triggered mEPAs (physical activity, screen time and sedentary behavior) in minutes.

Compliance	Phase	Mean	ANOVA (*p*-value)*	Tukey *post-hoc* test					
				Phase 1 *vs* Phase 2 (*p*-value)	Phase 1 *vs* Phase 3 (*p*-value)	Phase 1 *vs* Phase 4 (*p*-value)	Phase 2 *vs* Phase 3 (*p*-value)	Phase 2 *vs* Phase 4 (*p*-value)	Phase 3 *vs* Phase 4 (*p*-value)
mEPA (response/day)	Phase 1	3.06	21.370 (*p* < 0.001)	1.7455 (*p* < 0.001)	1.558 (*p* < 0.001)	1.981 (*p* < 0.001)	−0.188 (*p* = 0.937)	0.236 (*p* = 0.946)	0.423 (*p* = 0.751)
	Phase 2	1.31							
	Phase 3	1.50							
	Phase 4	1.08							
Time taken to respond to mEPAs (minutes/day)	Phase 1	117.37	4.69 (*p* = 0.003)	−50. 658 (*p* = 0.295)	−61.776 (*p* = 0.130)	−138.123 (*p* = 0.004)	−11.118 (*p* = 0.986)	−87.465 (*p* = 0.201)	−76.347 (*p* = 0.306)
	Phase 2	168.03							
	Phase 3	179.15							
	Phase 4	255.49							

**Notes:**

Weekly mEPAs were excluded from these analyses due to incompatible survey trigger periods (daily *vs* weekly), and insufficient sample size.

* Degrees of Freedom: F(3, 144).

Comparing Phase 1 and Phase 3, the mEPA responses (difference = 1.558, *p* < 0.001) indicate a significant difference between these phases. Specifically, the positive difference indicates that the responses received in Phase 1 are significantly greater than Phase 3. Similarly, the negative difference indicates that the time taken to respond in Phase 1 is lower than Phase 3. Additionally, the positive difference indicates that the average number of questions answered in Phase 1 is significantly greater than in Phase 4. The negative difference suggests that the average time taken to respond to mEPAs in Phase 1 is lower than in Phase 4.

The Tukey *post hoc* test comparing Phase 2 *vs* Phase 3 mEPA response (difference = −0.188, *p* = 0.937) and the average time taken to respond to mEPAs (difference = −11.118, *p* = 0.986) indicates no significant difference between Phase 2 and Phase 3. However, the positive difference indicates that the number of mEPA responses in Phase 3 is significantly greater than Phase 4. Finally, the negative difference indicates that the time taken to respond to mEPAs in Phase 3 is not significantly lower than Phase 4. These results illustrate the interplay between consistency, personalization, and user behavior within digital health platforms, highlighting the potential of HCI-based nudging strategies to optimize compliance.

## Discussion

This quasi-experimental study empirically investigated a serendipitous natural experiment that enabled assessment of varying digital nudging protocols on citizen compliance to a longitudinal mental health intervention. The study implemented a human-centered digital health platform that was central to the nudging protocol. The overall findings indicate that HCI-based nudging through an adaptive digital health platform may be associated with citizen compliance, with a potential dose-response relationship between human-triggered nudges (*i.e*., scientist-triggered) and compliance of youth, irrespective of the consistency of computer-triggered nudges (*i.e*., system-triggered).

To our knowledge, this study is the first to assess nudging practices on participant compliance within a longitudinal intervention that integrates health and education systems to promote youth mental health. This integration is especially crucial in rural and Indigenous contexts, where health and educational services often operate in silos and institutional trust may be strained due to histories of marginalization (Nguyen et al., 2020; Larson et al., 2024). By embedding a mental health intervention within the school environment and enabling continuous engagement through a digital platform, our study demonstrates how such integration can create more accessible, trusted, and culturally relevant pathways for care delivery.

In particular, the application of citizen science *via* a digital health platform provides a pathway for systems integration (Katapally, 2020b). Digital citizen science can empower citizens to actively participate in scientific research using personally-owned ubiquitous devices (*i.e*., smartphones) through active contribution, co-design, or co-creation of digital health platforms (Katapally, 2020a, 2019, 2020b; Ibrahim, Hammami & Katapally, 2023). In our study, youth contributed to the design and timing preferences of nudging protocols, as well as offered feedback on platform usability throughout implementation. This direct involvement likely enhanced the personal relevance and perceived ownership of the intervention (Newman et al., 2017; Rotman et al., 2014), increasing responsiveness to communications and strengthening the observed dose-response relationship between human-triggered nudges and compliance.

For instance, personalized nudges like the “Best Picture” feature were designed to reflect youth-generated content, reinforcing the reciprocal and participatory nature of the intervention. As such, digital citizen science not only supported personalized engagement, but also appeared to amplify behavioral responsiveness, suggesting that co-designed, human-centered digital interventions can more effectively sustain participation in remote interactions with citizens (Malloy et al., 2023; Kilfoy et al., 2024). Moreover, digital citizen science approaches can facilitate systems integration by ethically leveraging big data that exist outside traditional health systems using citizen-owned ubiquitous devices (Katapally, 2019)—a current gap that limits current health systems to provide timely (Pastorino et al., 2019) for urgent issues including the youth mental health crisis that requires rapid responses (Colizzi, Lasalvia & Ruggeri, 2020).

### Natural experiment-enabled nudging phases

The serendipitous natural experiment was unplanned, as the phased nudging protocol was enabled by cloud computing system errors that were beyond the control of the research team. This natural experiment resulted in four study phases with varied consistency and implementation of three nudging protocols: (1) System-triggered notifications that automatically asked youth to complete ecological assessments capturing their daily physical activity and sedentary behavior. (2) Non-personalized scientist-triggered notifications triggered every Friday asking youth to provide input about the impact of land-based activities on their mental health (*i.e*., the intervention). (3) Personalized scientist-triggered notifications titled “Best Picture” that were triggered every Monday to showcase the best images submitted by youth from the previous week, which highlighted their compliance and encouraged them to continue their involvement in the land-based activities (*i.e*., the intervention).

We found significant differences in youth compliance levels throughout most study phases. Consistent, uninterrupted nudging in Phase 1, including both system-triggered, and non-personalized and personalized scientist-triggered nudges, resulted in the highest youth compliance as well as fastest response times. While this pattern aligns with our hypothesis about the benefits of comprehensive nudging, it is important to interpret these findings as associative rather than causal. Other time-dependent factors may have contributed to the observed differences across phases. For instance, higher engagement in Phase 1 was partially driven by the novelty of the platform and study participation itself. This aligns with literature on the novelty effect, which suggests that participant responsiveness often peaks in early stages of digital interventions before declining as the new experience becomes routine or less stimulating (Shin et al., 2019). Seasonal variation, evolving participant-researcher rapport, and changes in school routines or peer dynamics may also have played a role in shaping compliance behavior over time. Despite these potential confounders, the phase *vs* phase independent comparisons suggest that the consistency of human-triggered nudges could be linked with higher and faster response rates from youth. However, this finding should be carefully interpreted to account for the lack of significant difference in youth compliance between phase 2 *vs* phase 3. Existing evidence suggests that the receipt of any message of any type in the form of nudges influences human behavior (Alm et al., 2019), particularly if the nudges are momentary reminders in real-world settings (Hyytinen, Tuimala & Hammar, 2022). Our study’s findings point towards a key nuance—consistency of human-triggered digital nudges is critical for participant response rates as well as response times, which are primary indicators for facilitating real-time interactions. In our study, this held true for daily time-triggered mEPAs, as well as citizen-triggered mEPAs, but responses continued to diminish for the weekly land-based activities mEPAs. The highest response rate for weekly mEPAs was during Phase 1 while nudging was consistent, however the response rate could not be sustained in other phases, even with the reintroduction of consistent nudging in Phase 3. This corroborates existing evidence that weekly nudging may not be enough to significantly change participant attitude or behavior (Taback & Gibbs, 2023; Georgette et al., 2017; Mbuagbaw et al., 2012). Instead, more frequent nudges, like daily reminders, are needed to see significant changes (Chen et al., 2017; Horsch et al., 2017; Balato et al., 2013; Belton et al., 2013; Strandbygaard, Thomsen & Backer, 2010; Armstrong et al., 2009). It is important to acknowledge that the intervention was implemented within an educational setting, and the school-based land-based programs were delivered by educators as part of regular curricula. While these programs were designed independently of the digital nudging protocol, the surrounding school environment—including teacher facilitation, classroom norms, and peer dynamics—may have indirectly influenced how youth engaged with the app. However, educators did not interact with the digital platform and were not involved in the triggering, timing, or personalization of any nudges. This design minimizes the likelihood of direct educator influence on the digital compliance metrics observed in this study.

Nudging has been an effective tool for behavior change across contexts and domains, including health, environmental, prosocial, behavioral, financial and food domains (Mertens et al., 2022), though its effectiveness remains unclear specifically in digital health interventions to increase compliance and adherence. Some studies suggest that digital nudging improves compliance and responsiveness to interventions, which ultimately increases the effectiveness of digital health platforms (Purohit et al., 2023; Haile et al., 2020). However, complex public health issues such as non-communicable disease prevention may require a multifaceted approach. While nudging can contribute to behavior change linked to non-communicable disease prevention, evidence suggests that it should be used alongside traditional approaches, such as awareness raising, financial incentives, and regulations to address complex public health issues (Murayama et al., 2023; Ewert, 2020). One aspect that seems to improve nudging effectiveness is consistency (*i.e*., continuous engagement), potentially due to nudging aiding with habit formation (Byrne et al., 2024). Our study indicates that employing consistent nudges during Phase 1 potentially aided in the development of a habit of compliance with the mental health intervention. Once consistent nudges were interrupted during Phase 2 and compliance levels decreased significantly, it was challenging to raise compliance to the original levels, even after reintroducing consistent nudging during Phase 3—a phenomenon that potentially transpired due to loss of habit formed through the consistent and interactive digital nudging system in Phase 1 (Olson et al., 2022).

### Personalized digital nudging

Two broad types of digital nudging were used in this study: system-triggered and scientist-triggered interactions. System-triggered (*i.e*., automated time-triggered) nudges have been shown to provide timely and consistent engagement during study trials (Kay & Bostock, 2023; Motz, Mallon & Quick, 2021). However, our findings reinforce a growing body of evidence suggesting that personalized scientist-triggered nudges may offer greater impact by fostering a sense of relevance and interpersonal connection (Newman et al., 2017; Rotman et al., 2014; de Vries, Bol & van der Laan, 2025). This distinction became particularly evident in the engagement trends observed between Phases 2 and 3. For instance, youth engagement slowed down initially, and then completely stopped in Phase 2 during which no personalized scientist-triggered nudges were sent. Nevertheless, levels of two mEPA metrics, physical activity and “Take a Picture”, which were zero in Phase 2, increased to 15 and 6 respectively in Phase 3 following the reintroduction of personalized scientist-triggered nudges.

Once personalized scientist-triggered nudges were interrupted again in Phase 4, even with consistent non-personalized scientist-triggered nudges, levels of the same mEPA metrics dropped to 3 for physical activity and 2 for “Take a Picture”. Sedentary and screen time mEPA responses also dropped from 36 in Phase 3 to 11 in Phase 4. These findings align with existing evidence suggesting that human-generated nudges based in HCI principles—particularly feedback, user-centered design, and transparency—often resonate more deeply with users due to their personalization and responsiveness to real-time data and user interaction (Bergram et al., 2022; Mele et al., 2021; Shin & Kim, 2018; Katapally, Hammami & Chu, 2021). The “Best Picture” nudges served as visual feedback loops, showing youth that their contributions were seen and valued. This kind of system responsiveness and reinforcement highlights how user-centered and transparent interfaces can sustain motivation. These findings also align with research in other disciplines, such as public e-services uptake, where nudges doubled adoption rates of underutilized digital services among slow adopters; cybersecurity, where personalized nudges tailored to individual decision-making styles significantly improved behavioral outcomes as compared to a “one-size-fits-all” approach; and learning environments, where reminder nudges enhanced study consistency and academic performance by shifting study behavior and increasing cognitive effort (Peer et al., 2020; Hyytinen, Tuimala & Hammar, 2022; O’Connell & Lang, 2018).

For instance, a study by Hyytinen, Tuimala & Hammar (2022) utilized “social norm nudges” (*i.e*., driving adoption through showcasing how peers behave) and found that social influence enhanced external motivation of participants to also adopt an online service. This personalization is similar to the “Best Picture” personalized scientist-triggered nudge that was sent in our study, which showcased the best picture taken by youth—a nudge that could have enhanced external motivation of other youth in the study. The role of personalization within human-triggered nudges is also evident from the fact that there were no significant differences in compliance between Phase 2 and Phase 4—both these phases received almost no personalized nudges even though consistent non-personalized scientist nudges were sent during Phase 4. Moreover, there was a significant decrease in compliance from Phase 3 to Phase 4, when the only key difference between these phases was consistent personalized nudging in Phase 3 *i.e*., compliance decreased between these phases even though the non-personalized scientist nudges were sent consistently.

The nudges administered to participants in our study were personalized using a pool of citizen-generated data and the same nudge was sent to all participants to enable external motivation. However, studies have shown the potential of integrating digital phenotyping in mental health interventions to refine personalization and utilize behavioral differences in individuals during the study period (Oudin et al., 2023; Ibrahim et al., 2021; Bernardos et al., 2019). Defined as the observation of differing participant behavior, digital phenotyping has been used to design and administer personalized nudges based on individual sensor and interaction data (Ibrahim et al., 2021). This type of highly personalized nudging, which is dependent on existing individual compliance, holds promise to take our nudging approach a step further to increase youth compliance. For example, administering individual-specific nudges with different messaging and at different frequencies based on whether youth are actively participating or not to promote greater engagement. However, the application of digital phenotyping also raises important ethical considerations, including concerns about informed consent, data privacy, and the risk of algorithmic bias (Martinez-Martin, Greely & Cho, 2021; D’Alfonso et al., 2025; Mulvenna et al., 2021). These challenges must be addressed through transparent design, rigorous validation, and community governance before such technologies can be responsibly scaled.

### The future of digital health platforms

This study consisted of a digital health platform featuring two key HCI interfaces aimed to improve remote human-to-human engagement—a citizen-facing smartphone app and a scientist-facing dashboard. In essence, citizens interacted with the app to submit data and receive nudges, while researchers monitored engagement and triggered personalized messages *via* the dashboard in near real-time. These interactions enabled a dynamic feedback loop in which real-time data informed adaptive engagement strategies.

Digital nudging in this context was integrated with integrated knowledge translation—a health research approach that emphasizes collaboration with knowledge users (*e.g*., patients, practitioners, communities) across all stages of the research process, from design to dissemination. These data were used by scientists to enable digital nudging, and in turn, digital nudging was utilized for integrated knowledge translation—a health research approach that emphasizes collaboration with knowledge users (*i.e*., patients, practitioners, communities) across all stages of the research process, from design to dissemination (Lawrence, Bishop & Curran, 2019)—a novel strategy of combining digital nudging with integrated knowledge translation. In this study, Indigenous youth were engaged as knowledge users through co-design of intervention activities, customization of digital features, and ongoing feedback mechanisms. Nudging was not only a tool for promoting compliance, but also a mechanism for real-time knowledge exchange, reflecting integrated knowledge translation principles by enabling participants to influence the intervention in real time based on their own data.

Digital health platforms can transform integrated knowledge translation by enabling real-time engagement with citizens to potentially link translation of research findings with behavior changes *via* digital nudging (Katapally, 2020a, 2020b; Katapally, Hammami & Chu, 2021). Research suggests that the presence of a human element significantly influences data collection outcomes, evidence that not only aligns with our study’s findings, but also provides some direction for use of AI in digital health platforms. Since our findings indicate that personalized, human-triggered nudges were key to sustaining youth engagement, future AI-driven systems should aim to augment, rather than replace, human interaction. For example, Mou & Xu (2017) found that users tend to be more open, agreeable, and self-disclosing when interacting with humans rather than AI. As one of the fastest growing and increasingly popular data-driven technologies (Said et al., 2023), AI is becoming a standard in many contexts (Xu et al., 2021). Beyond mere presence, the incorporation of specific human characteristics into human-AI interaction has been shown to further enhance user experiences. Studies have also demonstrated that integrating empathetic characteristics into AI interfaces can lead to increased acceptance and trust between consumers and brands (Pelau, Dabija & Ene, 2021; Kim & Im, 2023). Similarly, the inclusion of emotional cues, such as a sense of humor, into human-AI interaction has been found to increase closeness and interpersonal attraction and promote friendship and trust between users and AI (Xie et al., 2023). However, these benefits must be balanced with ethical considerations. Designing AI to mimic human behavior too closely may risk deception or manipulation (Kafali et al., 2024; John, 2025), particularly if users are unaware they are interacting with a machine. Thus, safeguards should be implemented to ensure transparency, maintain user trust, and promote the ethical use of human-like AI in sensitive contexts such as youth mental health.

To optimize citizen engagement and data quality in health interventions, digital health platforms can leverage human-centered AI approaches to reduce the perceived distance between humans and computers. Studies suggest embedding various personalities and responses in computers based on user needs and social cues (Mou & Xu, 2017; Williams, Fiore & Jentsch, 2022), which can enhance the user experience of human-computer communication. This personalized approach, driven by actual human input, holds potential for improving the implementation of health interventions using digital health platforms (Williams, Fiore & Jentsch, 2022). By building systems that closely mimic human-to-human interaction, digital health platforms can enhance citizen/patient experiences and obtain better data for more effective health interventions.

### Key recommendations

Based on the findings from this study, several key recommendations can be made for the development and implementation of digital health platforms to enhance participant engagement and compliance in health interventions.

(1) Applying digital citizen science: Incorporate citizen science into digital health platforms to empower participants as co-creators of knowledge. Enable participants to contribute data using personally-owned digital devices to foster a sense of ownership and engagement (Katapally, 2020a, 2020b; Katapally, Hammami & Chu, 2021).
(2) Consistent HCI-based nudging practices: Implement consistent nudging strategies rooted in HCI principles throughout interventions to maintain participant engagement and compliance. Utilize automated nudges for timely and consistent engagement, complemented by personalized human-triggered nudges to foster deeper connections (Kay & Bostock, 2023; Bergram et al., 2022).
(3) Personalization of nudging: Personalize nudging approaches based on individual behavior and responses to the digital platform. Utilize digital phenotyping techniques to observe and understand participant behavior, allowing for the design and administration of personalized nudges tailored to individual needs and preferences (Oudin et al., 2023; Ibrahim et al., 2021; Bernardos et al., 2019).
(4) Incorporation of human elements: Incorporate human elements, such as empathy and humor, into user interfaces to increase user acceptance, trust, and engagement. Build systems that acknowledge individual nuances and foster a sense of connection in digital health platforms to enhance user satisfaction (Pelau, Dabija & Ene, 2021; Kim & Im, 2023; Xie et al., 2023).
(5) Human-centered AI approaches: Leverage human-centered AI approaches to reduce the perceived distance between humans and computers. Embed various personalities and responses in AI interfaces based on user needs and social cues to enhance the user experience of human-computer communication. Design AI interfaces that closely mimic human-to-human interaction to resonate with users on emotional and interpersonal levels. This approach can lead to enhanced user experiences and better data collection outcomes, ultimately contributing to more effective health interventions (Mou & Xu, 2017; Williams, Fiore & Jentsch, 2022).
(6) Prioritization of digital literacy: Integrate comprehensive digital literacy programs into digital health platforms to further aid with compliance, while mitigating potential risks associated with technology use (Buchan, Bhawra & Katapally, 2024; Fitzpatrick, 2023).
(7) Digital health evaluation: Incorporate iterative evaluation approaches to inform evidence-based development of digital health platforms to continuously assess usability, functionality, and effectiveness for enhanced user engagement (Buchan, Katapally & Bhawra, 2024).

### Strengths and limitations

The primary strength of this study is its quasi-experimental design, which enabled real-time observation of behavior during an unplanned disruption in the nudging protocol. This natural experiment increased the ecological validity of the findings by reflecting real-world complexities and allowing for practical insights under imperfect implementation conditions. Through a digital citizen science approach, participants engaged through their own mobile devices across both iOS and Android platforms. Participants were located in rural and remote First Nations communities in western Canada, allowing for geographic and seasonal variation in responses and capturing diverse contexts in terms of connectivity, culture, and environment. Additionally, the complex deployment of three different nudge types, including system-triggered, scientist-triggered, and personalized scientist-triggered nudges, provided insights into participant compliance dynamics. System-triggered nudges aligned with habituation theory, providing consistent prompts to build behavioral routines. Non-personalized scientist-triggered nudges reflected reminder theory, reinforcing regular task completion without individual tailoring. Personalized scientist-triggered nudges incorporated principles from social proof and reinforcement learning by showcasing peer-generated content and rewarding participation with individualized recognition. These theoretical distinctions allowed for a nuanced analysis of compliance patterns and user engagement.

An inherent limitation in our study involves the susceptibility of digital platforms to breakdowns, which are inevitable. However, in this case, the interruption enabled a natural experiment framework by creating distinct “on” and “off” phases for specific nudging types—an opportunity that would have been difficult to ethically or logistically implement through planned manipulation. This allowed us to assess the impact of nudging consistency in a real-world setting. Limitations also include the small sample size, which reduced statistical power and increased the risk of overestimating effect sizes. Additionally, findings may not generalize beyond the study population due to the contextual specificity of the intervention (*i.e*., rural Indigenous youth in western Canada). The lack of data on reasons for non-compliance, particularly for weekly mEPAs, further limits interpretation. These weekly surveys had lower response rates, likely due to their longer format, diminished novelty over time, and lack of direct feedback or visible incentives tied to completion—unlike the more interactive and reinforced daily mEPAs. Although the same participants contributed responses across all four phases, uniformly low and uneven response counts precluded the use of repeated-measures ANOVA or mixed-effects models, as these would risk unstable estimates and inflated error rates. Consequently, responses were treated as independent, a conservative choice we acknowledge as a limitation, recommending future studies with larger and more balanced samples apply repeated-measures approaches. Another limitation is the short time frame of each phase, which may have also limited our ability to detect long-term engagement patterns or distinguish between short-term variability and sustained behavioral change. Engagement declines could reflect temporal fatigue rather than the direct absence of nudging protocols.

Future studies should consider larger, more diverse samples and longer intervention durations. Methodologically, mixed-effects models could help account for intra-individual changes over time, and adaptive trial designs may offer a way to dynamically test different nudging strategies in response to participant behavior. Additionally, qualitative methods such as interviews or in-app feedback could provide insights into user experience and non-compliance, improving design and personalization of future nudging systems.

## Conclusion

This is the first quasi-experimental study to assess a serendipitous natural experiment that resulted in varying digital nudging protocols, which in turn may have influenced youth compliance to a longitudinal mental health intervention that integrates health and education systems. Our findings emphasize the transformative potential of digital health platforms to bridge systems fragmentation to address existing limitations within health systems by enabling youth-driven, real-time feedback loops that support mental health promotion in culturally grounded, school-based settings. Personalized “Best Picture” nudges and consistent automated reminders emerged as the most effective in improving both the volume and speed of youth responses, highlighting the critical role of human-triggered personalization and temporal consistency in digital engagement strategies.

By utilizing digital citizen science, the study demonstrated both the technical feasibility and social acceptability of engaging empowered youth as active contributors (*i.e*., co-creators) to their own health interventions. This participatory model supports a scalable framework for future programs that aim to deliver ethical, community-driven mental health support in underserved and remote regions. The integration of tailored nudging within adaptive user interfaces highlights a pathway for enhancing interactivity and user-centered design in digital health systems—a finding that supports the need to incorporate human-centered AI in implementing future digital health platforms.

## Additional Information and Declarations

### Data Availability

The following information was supplied regarding data availability:

The anonymized data are available at figshare: Ibrahim, Sheriff; Katapally, Tarun Reddy (2024). Human-centered nudging to promote youth mental health: A serendipitous natural experiment enabled by a digital health platform. figshare. Dataset. https://doi.org/10.6084/m9.figshare.25632600.v1.
